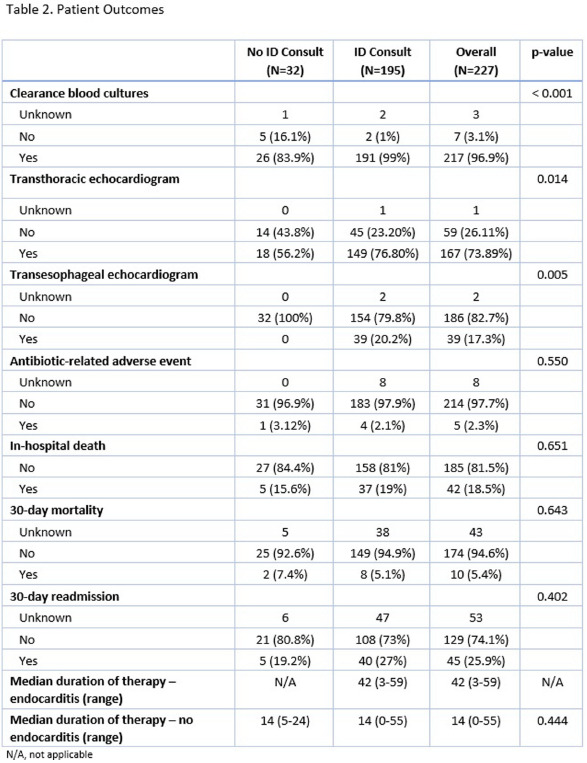# Impact of Infectious Diseases Consultation for Patients with Enterococcal Bacteremia: a Retrospective Cohort Study

**DOI:** 10.1017/ash.2025.258

**Published:** 2025-09-24

**Authors:** Conor Vinaixa, Haley Pritchard

**Affiliations:** 1 Indiana University School of Medicine; 2 Indiana University School of Medicine

## Abstract

**Background:** Gram-positive bacteremia is a challenging cause of morbidity and mortality. Past publications have shown improved patient outcomes and increased adherence to recommended standards of care with infectious disease consultation (IDC) for Staphylococcus aureus bacteremia1. Enterococcus species are another common cause of gram-positive bacteremia with significant morbidity and mortality. This study aims to assess the impact of IDC on the care of patients with Enterococcal bacteremia. **Methods:** A retrospective chart review was performed on 227 inpatients with at least one blood culture growing an Enterococcus species between June 2022 and November 2023. Patient characteristics collected included age, Charlson Comorbidity index, presence of endocarditis, source of bacteremia, and consultation of the inpatient ID service. Outcomes assessed included in-hospital and 30-day mortality, 30-day re-admission rate, acquisition of repeat blood cultures to document clearance of bacteremia, transthoracic (TTE) and/or transesophageal echocardiography (TEE), and anti-Enterococcal antibiotic duration. Categorical variables were compared with Chi-square or Fisher’s exact tests. Continuous variables were compared with independent t-tests or Mann-Whitney U nonparametric tests. **Results:** Of 227 patients, 195 (85.8%) received IDC while 32 (14.2%) did not. Patients in both groups had similar Charlson comorbidity indices. 23 (11.7%) patients had Enterococcal endocarditis, all of whom received IDC (Table 1). Patients with IDC had a significantly higher rate of acquisition of clearance blood cultures (98.96% vs. 83.87%, p 76.80% vs. 56.25%, p = .014), and TEE (20.21% vs 0.0%, P = .005) (Table 2). There were no significant differences in in-hospital mortality, 30-day mortality, 30-day re-admission rate, or duration of anti-Enterococcal antibiotics. **Conclusions:** These results support the conclusion that patients with Enterococcal bacteremia who received IDC were more likely to be managed according to currently recommended standards of care. In this cohort, IDC did not have a statistically significant association with differences in mortality, re-admission rate, or antibiotic duration. Patients with Enterococcal bacteremia are likely to benefit from IDC, especially as they frequently have significant life-limiting co-morbidities complicating their care. **References:** Vogel M, Schmitz RP, Hagel S, Pletz MW, Gagelmann N, Scherag A, Schlattmann P, Brunkhorst FM. Infectious disease consultation for Staphylococcus aureus bacteremia - A systematic review and meta-analysis. J Infect. 2016 Jan;72(1):19-28. doi: 10.1016/j.jinf.2015.09.037. Epub 2015 Oct 9. PMID: 26453841.

**Figure:**
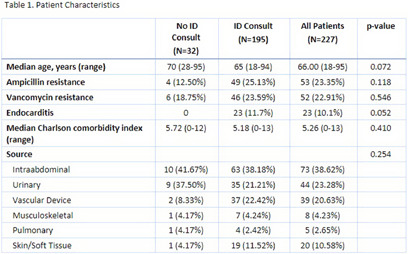

**Figure:**